# Molecular dissection of germline chromothripsis in a developmental context using patient-derived iPS cells

**DOI:** 10.1186/s13073-017-0399-z

**Published:** 2017-01-26

**Authors:** Sjors Middelkamp, Sebastiaan van Heesch, A. Koen Braat, Joep de Ligt, Maarten van Iterson, Marieke Simonis, Markus J. van Roosmalen, Martijn J. E. Kelder, Evelien Kruisselbrink, Ron Hochstenbach, Nienke E. Verbeek, Elly F. Ippel, Youri Adolfs, R. Jeroen Pasterkamp, Wigard P. Kloosterman, Ewart W. Kuijk, Edwin Cuppen

**Affiliations:** 1 Center for Molecular Medicine and Cancer Genomics Netherlands, Division Biomedical Genetics, University Medical Center Utrecht, Universiteitsweg 100, Utrecht, 3584CG The Netherlands; 2 Cardiovascular and Metabolic Sciences, Max-Delbrück-Center for Molecular Medicine (MDC) in the Helmholtz Association, Robert-Rössle-Strasse 10, Berlin, 13125 Germany; 3 Department of Cell Biology, Center for Molecular Medicine and Regenerative Medicine Center, University Medical Center Utrecht, Uppsalalaan 6, Utrecht, 3584CT The Netherlands; 4 Department of Molecular Epidemiology, Leiden University Medical Center, Einthovenweg 20, Leiden, 2333ZC The Netherlands; 5 Cergentis B.V., Yalelaan 62, Utrecht, 3584CM The Netherlands; 6 MRC Human Genetics Unit, Institute of Genetics and Molecular Medicine, University of Edinburgh, Crewe Road, Edinburgh, EH4 2XU UK; 7 Department of Pediatric Pulmonology & Laboratory of Translational Immunology, Wilhelmina Children’s Hospital, University Medical Centre, Lundlaan 6, Utrecht, 3584EA The Netherlands; 8 Department of Genetics, University Medical Center Utrecht, Lundlaan 6, Utrecht, 3584EA The Netherlands; 9 Department of Translational Neuroscience, Brain Center Rudolf Magnus, University Medical Center Utrecht, Universiteitsweg 100, Utrecht, 3584CG The Netherlands

**Keywords:** Chromothripsis, Complex genomic rearrangements, Congenital disorders, Induced pluripotent stem cells, Neuronal differentiation, RNA-sequencing, Chromosome conformation capture, *TWIST1*, Craniosynostosis, Personal genomics

## Abstract

**Background:**

Germline chromothripsis causes complex genomic rearrangements that are likely to affect multiple genes and their regulatory contexts. The contribution of individual rearrangements and affected genes to the phenotypes of patients with complex germline genomic rearrangements is generally unknown.

**Methods:**

To dissect the impact of germline chromothripsis in a relevant developmental context, we performed trio-based RNA expression analysis on blood cells, induced pluripotent stem cells (iPSCs), and iPSC-derived neuronal cells from a patient with de novo germline chromothripsis and both healthy parents. In addition, Hi-C and 4C-seq experiments were performed to determine the effects of the genomic rearrangements on transcription regulation of genes in the proximity of the breakpoint junctions.

**Results:**

Sixty-seven genes are located within 1 Mb of the complex chromothripsis rearrangements involving 17 breakpoints on four chromosomes. We find that three of these genes (*FOXP1*, *DPYD*, and *TWIST1*) are both associated with developmental disorders and differentially expressed in the patient. Interestingly, the effect on *TWIST1* expression was exclusively detectable in the patient’s iPSC-derived neuronal cells, stressing the need for studying developmental disorders in the biologically relevant context. Chromosome conformation capture analyses show that *TWIST1* lost genomic interactions with several enhancers due to the chromothripsis event, which likely led to deregulation of *TWIST1* expression and contributed to the patient’s craniosynostosis phenotype.

**Conclusions:**

We demonstrate that a combination of patient-derived iPSC differentiation and trio-based molecular profiling is a powerful approach to improve the interpretation of pathogenic complex genomic rearrangements. Here we have applied this approach to identify misexpression of *TWIST1*, *FOXP1*, and *DPYD* as key contributors to the complex congenital phenotype resulting from germline chromothripsis rearrangements.

**Electronic supplementary material:**

The online version of this article (doi:10.1186/s13073-017-0399-z) contains supplementary material, which is available to authorized users.

## Background

Disruption of the genomic architecture by structural rearrangements such as translocations, deletions, duplications, and inversions is an important cause of congenital disease [1]. It has been estimated that approximately 15% of patients with multiple congenital abnormalities and/or mental retardation (MCA/MR) have a clinically relevant structural genomic rearrangement [2–5]. Some of these patients have very complex combinations of structural variants resulting from chromothripsis, the local shattering and reassembly of one or a few chromosomes in a single event [6–8]. Chromothripsis can occur in both somatic cells, where it can contribute to cancer, and germline cells, where it can lead to congenital disorders [6, 9, 10]. Congenital chromothripsis cases with up to 57 breakpoints involving one to five chromosomes have been described [7, 11]. Determining the molecular and phenotypic consequences of genomic rearrangements is a major challenge, especially for patients with complex rearrangements that involve large genomic regions of several megabases on multiple chromosomes containing many genes and regulatory elements [12, 13]. Structural rearrangements may lead to altered gene expression, gene fusions, disruption of regulatory elements such as enhancers and boundaries of topologically associated domains (TADs), and/or unmasking of recessive mutations in the unaffected allele [12–16]. Due to the large number of potentially affected genes in patients with complex rearrangements, the molecular mechanisms that have contributed to their congenital phenotypes are often unknown. Transcriptome analysis is a powerful method to determine the functional molecular consequences of structural rearrangements [17–20]. Patients’ blood cells are commonly used as the source for RNA-seq analysis because of the relatively easy accessibility of this material. However, genes potentially involved in the disease of a patient may be expressed differently or not at all in blood compared to the disease-relevant tissue [21, 22]. In addition, congenital disorders are typically the result of defects in developmental programs and it is questionable whether deregulation of developmental gene expression patterns persists in adult tissues. One approach that circumvents these concerns is to recapitulate certain developmental processes by generating induced pluripotent stem cells (iPSCs) from patients and differentiate these towards disease-relevant cell types [23–25]. This strategy has been applied successfully to improve our understanding of the molecular mechanisms underlying several (neuro-)developmental diseases such as schizophrenia and Rett syndrome [26, 27].

We previously performed RNA-seq on blood samples of patients with germline chromothripsis and identified several molecular phenotypes caused by the genomic rearrangements [19]. These included a hyper-activated trophoblast-specific miRNA cluster that interferes with embryonic brain development when ectopically expressed [19]. However, in a second patient with MCA/MR the relevance of the identified molecular effects to the phenotype could not be entirely resolved due to the complexity of the rearrangements [19]. In this study we further dissected the molecular consequences of chromothripsis by analyzing RNA expression and genome architecture in disease-relevant cell types derived from iPSCs from this patient and both parents.

## Methods

### Derivation and cultivation of iPSCs

Peripheral blood samples were obtained from a family trio consisting of the patient (child) with germline chromothripsis and both parents who served as controls. Peripheral blood mononuclear cells (PBMCs) were isolated by separation on a Ficoll-Paque TM PLUS gradient (GE Healthcare) with a density of 1.077 g/ml. Subsequently, CD34-positive cells were magnetically labeled with CD34-microbeads and purified with a CD34 Microbead kit (Miltenyi). The purified CD34-positive cells were resuspended in PBMC medium consisting of Iscove’s modified Dulbecco’s medium (ThermoFisher Scientific) with 5% fetal calf serum, 50 ng/ml stem cell factor, 50 ng/ml FLT3-ligand, 50 μM β-mercaptoethanol, 10 μg/ml penicillin, 10 μg/ml streptomycin, and 2 mM L glutamine, and plated in flat bottom 96-well ultra-low attachment plates. After 5 days, cells were passaged and the PBMC medium was further supplemented with 20 ng/ml interleukin (IL)-6 and 20 ng/ml thrombopoietin (TPO). After 7 days, cells were spin-transduced with 1 ml OSKM-dTOMATO lentivirus [28] supplemented with 8 μg/ml polybrene, 50 ng/ml stem cell factor, 50 ng/ml FLT3-ligand, 20 ng/ml IL-6, and 20 ng/ml TPO at 1800 rpm at 32 °C for 100 minutes. Cells were subsequently incubated for 3 h, after which medium was changed to PBMC medium supplemented with IL-6 and TPO. The spin-transductions were repeated at day 9 and day 10 and cultures continued in PBMC medium supplemented with IL-6 and TPO. Subsequently all cells were seeded on irradiated mouse embryonic fibroblasts (Amsbio) and cultured in human embryonic stem cell (hESC) medium consisting of DMEM-F12 supplemented with 20% knock-out serum replacement, 10 μg/ml penicillin, 10 μg/ml streptomycin, 2 mM L-glutamine, 0.1 mM MEM-NEAA, 0.1 mM β-mercapthoethanol, and 10 ng/ml basic fibroblast growth factor. The hESC medium was refreshed daily. Three clonal iPSC lines were derived from the patient, two lines from the father and one from the mother. The iPSCs were subsequently adapted to and cultured on Geltrex-coated plastic (ThermoFisher Scientific) in serum- and feeder-free Essential-8 medium (ThermoFisher Scientific) with 1× penicillin-streptomycin (ThermoFisher Scientific). All cell lines were free of mycoplasm contamination.

### Differentiation of iPSCs towards the neural lineage

Differentiation of the iPSCs to neural progenitors was performed according to the protocol by Shi et al. [29] with several modifications. iPSCs were prepared for neural induction by culturing cells in three wells of a six-well plate to 90% confluency on Vitronectin-coated plates using the Essential-8 medium, after which cells were passaged in a 1:2 ratio to Geltrex-coated six-well plates. Cells were then cultured until 95–100% confluency, upon which the medium was switched to neural induction medium. Neural induction medium was prepared with a 1:1 mixture of DMEM/F-12-Glutamax (Life Technologies) and Neurobasal medium (Life Technologies) with added 1× N-2 supplement (Life Technologies), 1× B-27 supplement (Life Technologies), 5 μg/ml insulin (Sigma), 2 mM L-glutamine (Life Technologies), 1× non-essential amino acids (Life Technologies), 100 μM β-mercaptoethanol (Life Technologies), 1 μM dorsomorphin (Sigma), and 10 μM SB431242 (Tocris Bioscience). Medium was replaced daily. RNA was collected at days 0, 7, and 10 of differentiation. At day 10, cells were passaged to laminin-coated coverslips for later immunofluorescent staining. Medium was then switched to neural maintenance medium (neural induction medium without dorsomorphin and SB431242), in which cells were cultured until formation of neural rosettes on day 15 after neural induction.

### Immunofluorescent labeling of cultured cells

For immunofluorescent staining, cells were grown on coverslips, after which they were fixed in 4% paraformaldehyde for 15 minutes at room temperature (RT). Coverslips were then washed briefly in PBST (90% phosphate-buffered saline (PBS), 10% fetal bovine serum (FBS), 0.05% Triton X-100), permeabilized in permeabilization buffer (90% PBS, 10% FBS, 0.5% Triton X-100) for 15 minutes and blocked in PBST at RT for 1 h. Cover slips were incubated with primary antibody solution at RT for 1 hr. Primary antibodies were diluted in PBST to a concentration of 2 μg/ml. The primary antibodies used were mouse anti-NANOG (MABD24, EMD Millipore), Goat anti-OCT3/4 (sc-8628, Santa Cruz), Rabbit anti-SOX2 (AB5603, Chemicon), and Goat anti-PAX6 (PRB-278P-100, Covance Inc.). The coverslips were then washed three times with PBST at RT for 10 minutes. Next, the secondary antibody diluted in PBST to a concentration of 2 μg/ml was added and the samples were incubated in the dark at RT for 1 h. Secondary antibodies used are donkey anti-rabbit 488 (A-21206, Invitrogen), donkey anti-goat 568 (A-11057, Invitrogen), goat anti-mouse 633 (A-21050, Invitrogen) and rabbit anti-goat 488 (A-11055, Invitrogen). The coverslips were again washed three times with PBST at RT for 10 minutes. Finally, the coverslips were mounted using 3 μl Vectashield mounting medium with DAPI (H-1200, Vectorlabs), after which fluorescence was detected by confocal microscopy (Leica TCS SPE). The same acquisition settings were used for all samples throughout each experiment.

### RNA extraction and sequencing

Samples for RNA sequencing were collected at days 0, 7, and 10 of neural differentiation of cell lines UMCU14 and UMCU15 from the patient, UMCU30 from the mother, and UMCU23 (with technical replicate) and UMCU32 from the father. RNA extraction was performed with Trizol (Life Technologies) according to the manufacturer’s protocol. The isolated RNA was poly(A) selected with the MicroPoly(A) Purist Kit (Life Technologies) and a subsequent CAP-selection was performed with the mRNA ONLY Eukaryotic mRNA isolation kit (Epicentre/Illumina). Next, the RNA was heat sheared followed by hybridization and ligation to the SOLID adapters according to the SOLID sequencing protocol. The RNA was subsequently reverse transcribed using the SOLID RT primer. After size-selection of the complementary DNA, it was amplified using a SOLID PCR primer and a unique barcoding primer for each library. Samples were sequenced on a SOLID Wildfire. RNA sequencing of patient and parental blood samples was performed previously [19].

### Analysis of RNA sequencing data

Reads were mapped to the human reference genome (GRCh37/hg19) using Burrows-Wheeler Aligner (BWA) [30]. The R package GenomicAlignments v1.6.3 was used to count reads overlapping exons [31]. DESeq v1.22.1 was used to normalize read counts for library size and differential expression was calculated using the DESeq nBinomtest function [32]. Hierarchical clustering based on the expression of the 500 genes with highest variance between all iPSC and neural progenitor cell (NPC) samples was performed using heatmap.2 from the gplots R package v2.17.0 (https://cran.r-project.org/web/packages/gplots/). Expression profiles of day 7 and day 10 NPCs clustered together and were therefore merged for downstream analysis (Additional file 1: Figure S1). Genes with more than ten normalized counts were considered expressed genes. Molecular effects were defined as gene expression differences of at least twofold between patient and parents. Circos plots for data visualization were generated using Circos software [33].

### Hi-C data generation and analysis

iPSC-derived NPCs of the patient (lines UMCU14 and UMCU15) and the father (UMCU23 and UMCU32) were crosslinked with 2% formaldehyde for 10 minutes. The crosslinking reaction was quenched by 0.125 M glycine. Following the crosslinking procedure, samples were centrifuged at 400 g at 4 °C for 8 minutes. Pelleted cells were washed with PBS and centrifuged again at 400 g at 4 °C for 5 minutes. Cells were lysed in 1 mL freshly prepared lysis buffer (50 mM Tris pH 7.5, 150 mM NaCl, 5 mM EDTA, 0.5% NP-40, 1% Triton X-100, and 1× complete EDTA-free Protease Inhibitor Cocktail (Roche)) on ice for 10 minutes. Nuclei were washed twice in cold PBS after completion of the cell lysis.

Isolated and cross-linked NPC nuclei were digested with the DpnII restriction enzyme (New England Biolabs). Subsequently, the proximity ligation of interacting fragments was performed using T4 DNA ligase (Roche) to produce the 3C template, according to a previously described protocol by Simonis et al. [34]. After reverse crosslinking and precipitation, 10 μg of the template was sheared in microtubes (AFA Fiber Pre-Slit Snap-Cap 6 × 16 mm, 520045) using the Covaris S2 sonicator (1 cycle of 25 s; duty cycle 5%, intensity 3, 200 cycles per burst, frequency sweeping). Fragments that ranged in size from 500 to 1500 bp were selected using a 2% agarose gel. Size-selected fragments (1.1 μg) were used as the input for the TruSeq DNA Low Sample (LS) protocol (Illumina). Constructed libraries were size-selected using a LabChip XT DNA 750 Assay Kit (Caliper), resulting in libraries between 800 and 950 bp. These Hi-C libraries were sequenced in a paired-end manner on the Illumina HiSeq 2500, resulting in 2 × 100-bp reads. Sequenced read pairs were mapped independently using Burrows-Wheeler Aligner (BWA-0.7.5a; settings were bwa mem -c 100 -M) [30] to the human reference genome (hg19). Reads were further processed as previously described [35].

### 4C-seq

4C-seq libraries were generated from crosslinked iPSC-derived NPCs of the patient (lines UMCU14 and UMCU15) and the father (UMCU23 and UMCU32) as previously described [36]. DpnII was used as primary restriction enzyme and NlaIII as secondary restriction enzyme. We PCR amplified 1.6 μg of each 4C template for each of the viewpoints using the primers listed in Additional file 2: Table S1. The amplified 4C libraries were pooled, spiked with 30% Phi X 174 DNA, and sequenced on the Illumina NextSeq500 platform in paired-end mode. Data were processed as previously described [37]. The 4C-seq reads were normalized based on the total number of captured reads per sample. We analyzed 1.3 to 4.3 million mapped reads per viewpoint.

Locations of TADs in H1-hESC cells were determined by Dixon et al. [38] and obtained from http://promoter.bx.psu.edu/hi-c/download.html. Enhancer activity determined by expanded 18-state ChromHMM analysis of H1-derived NPCs (E007) and primary foreskin fibroblasts (E056) was obtained from the Roadmap Epigenomics Mapping Consortium (http://egg2.wustl.edu/roadmap/data/byFileType/chromhmmSegmentations/ChmmModels/core_K27ac/jointModel/final). The dataset for the primary foreskin fibroblasts (E056) was selected because these cells have the highest *TWIST1* RNA expression of all cell types analyzed by the Roadmap Consortium (data not shown).

### Molecular cloning

*CNTN3* was amplified from a *CNTN3*-containing plasmid (RG221979 Origene). An In Fusion cloning kit (Clontech) was used to clone the amplicon into an empty plasmid with a pCAG promoter. High expression and proper cellular localization of *CNTN3* were confirmed by transfection of the pCAG *CNTN3* plasmid into HEK293 cells followed by western blotting and immunofluorescence with an antibody that recognizes CNTN3 (AF5539; R&D Systems; data not shown).

### In utero electroporations of CNTN3 overexpression plasmids

Animal use and care was in accordance with institutional and national guidelines (Dierexperimentencommissie). At E14.5, pregnant C57Bl/6 mice were anesthetized using isoflurane (induction 3–4%, surgery 1.5–2%) and sedated with 0.05 mg/kg buprenorfin hydrochloride in saline. The abdominal cavity was opened and the uterine horns containing the embryos were carefully exposed. The lateral ventricles of the embryos were injected with linearized pCAG-*CNTN3* or control DNA (linearized Nes714tk/lacZ) vectors dissolved in 0.05% Fast Green using glass micro-pipettes (Harvard Apparatus). Nes714tk/lacZ was a gift from Urban Lendahl (Addgene plasmid #47614) [39]. pCAG-GFP was co-injected with the vectors to identify successfully electroporated cells. Developing cortices were targeted by electroporation with an ECM 830 Electro-Square-Porator (Harvard Apparatus) set to five unipolar pulses of 50 ms at 30 V (950-ms interval) using a platinum tweezer electrode holding the head (negative poles) and a third gold-plated Genepaddle electrode (positive pole) on top of the head (Fisher Scientific). Embryos were placed back into the abdomen and abdominal muscles and skin were sutured separately.

### Immunofluorescent staining and analysis of brain sections

In utero electroporated embryos were collected at E16.5 and heads were fixed in 4% paraformaldehyde and submerged in 30% sucrose followed by freezing in 2-methylbutane. Sections of 20 μm were cut on a cryostat, mounted on Superfrost Plus slides (Fisher Scientific), air-dried, and stored at −20 °C until used for immunofluorescence. The sections were then blocked with 3% bovine serum albumin in PBS and 0.1% Triton, followed by an overnight incubation in rabbit anti-GFP (A11122, ThermoFisher Scientific) diluted in blocking solution. After washing with PBS the sections were incubated in goat anti-rabbit 488 diluted in blocking solution. Finally, the sections were counterstained with Hoechst and embedded in Fluorsafe before mounting on the coverslips. Cortices were imaged using conventional confocal microscopy using a Zeiss confocal microscope. Adobe Illustrator was used to place consistent rectangles divided in eight equal square bins on top of the acquired images, so that bin 1 starts at the ventricle border of the tissue and bin 8 ends at the pial surface. The number of GFP-positive cells were counted in each bin and divided by the total amount of cells in the rectangle.

## Results

### Complex genomic rearrangements caused by chromothripsis in an MCA/MR patient

**Fig. 1:**
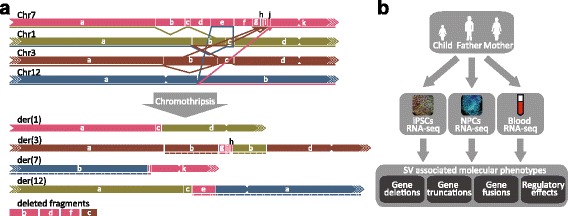
Overview of complex chromosomal rearrangements in the patient with MCA/MR and study design. **a** The breakpoint locations and genomic rearrangements on the four affected chromosomes in the germline chromothripsis patient determined by mate-pair and breakpoint fusion sequencing. Inversions are depicted with *dashed lines* beneath the derivative chromosomes. The four deleted fragments are shown below the derivative chromosomes. This illustration is adapted from van Heesch et al. [19]. **b** Overview of the experimental setup of this study. Molecular effects of the chromosomal rearrangements on deleted, truncated, and fused genes and genes within 1 Mb of the rearrangements were determined by trio-based RNA-seq of iPSCs and iPSC-derived neuronal cells from the patient and both parents. These data were compared with previously generated expression data of blood samples of the patient and parents to identify molecular phenotypes that contribute to the patient’s phenotype but are not detectable in blood [19]

Previously we performed RNA-seq on blood samples of an MCA/MR patient with germline chromothripsis and both parents. The phenotype of this patient includes craniosynostosis (premature fusion of one or more cranial sutures), facial dysmorphisms, duplication of the right thumb, pre- and postnatal growth retardation, and intellectual disability. Mate-pair and breakpoint junction sequencing showed that the genome of the patient contains 17 breakpoints on chromosomes 1, 3, 7, and 12 (Fig. 1a) [7]. Molecular phenotypes detected in blood could not entirely explain the patient's phenotype. Not all genes in proximity to the breakpoints were expressed in the patient’s blood samples, so we hypothesized that essential molecular effects that may have contributed to the patient phenotype were undetectable in the patient blood samples.

To obtain cell types relevant for the disease phenotype we generated three iPSC lines from the germline chromothripsis patient and differentiated two of these to the neural lineage (Fig. 1b). iPSCs were generated by reprogramming CD34-positive peripheral blood mononuclear cells (PBMCs) by transduction of a multicistronic lentiviral construct containing the canonical reprogramming factors [28, 40]. We also successfully generated two control iPSC lines from the father and one line from the mother. Karyotyping confirmed the presence of all four derivative chromosomes in the patient’s iPSC lines (Additional file 1: Figure S2). One of the patient’s cell lines contained a duplication of derivative chromosome 1 (Additional file 1: Figure S2b). The paternal lines contained normal chromosome numbers, but the cell line of the mother has a translocation between chromosome 20 and part of chromosome 1 (Additional file 1: Figure S2c). Because these karyotype abnormalities are distant from the breakpoints and because three of the five lines had the expected karyotypes, we concluded that these lines were suitable to study the effects of the rearrangements within 1 Mb of the breakpoints. All iPSCs expressed the pluripotency-associated factors OCT4, SOX2, and NANOG, as determined by immunofluorescence and western blotting (Additional file 1: Figure S3a, b). RNA-seq confirmed high expression of pluripotency factors in the iPSCs (Additional file 1: Figure S3c). Neural progenitor cells (NPCs) derived from the patient’s and parents’ iPSCs formed neural rosettes and expressed early neural markers such as *PAX6*, *OTX1*, *OTX2*, *SOX1*, and *SOX11* (Additional file 1: Figure S4).

### Molecular profiling of iPSC-derived neural progenitors

**Fig. 2:**
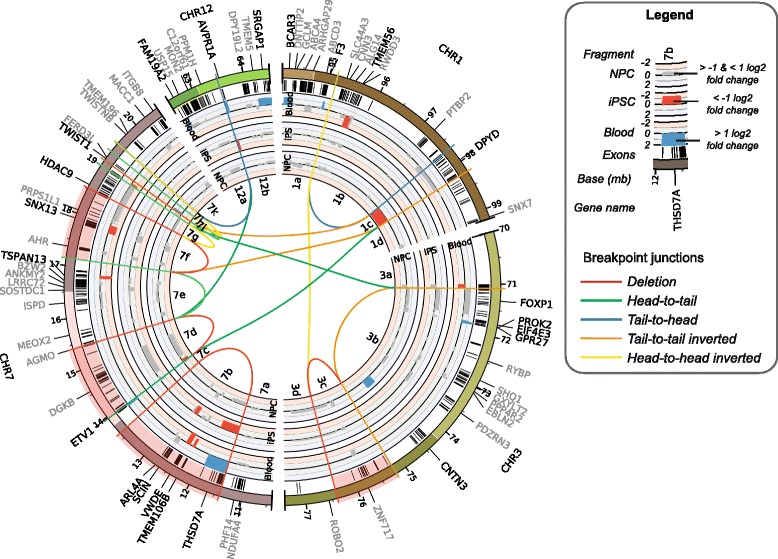
Impact of chromothripsis on expression of genes in proximity to rearrangements. Circos plot showing the regions affected by chromothripsis on patient chromosomes 1, 3, 7, and 12. The lines in the center of the plot visualize the 17 breakpoint junctions in the patient genome. In total, 67 genes, listed in the outer ring, are located on or within 1 Mb of the rearrangements. Exons are depicted as *black bars* beneath the chromosome ideograms. The *inside*, *center*, and *outside bar graphs* show the log2 fold expression differences (ranging from 2 to −2) between the patient and the parents in the iPSC-derived neural progenitors, the iPSCs, and the blood cells, respectively. Log2 fold expression differences of at least 1 between the patient and the parents are highlighted with *blue* (higher expression in patient) and *red* (lower expression in patient) *bars. Grey bars* indicate no or small (less than 1 log2 fold) expression differences between the patient and the parent. No bars are shown for genes with less than ten normalized read counts

**Fig. 3:**
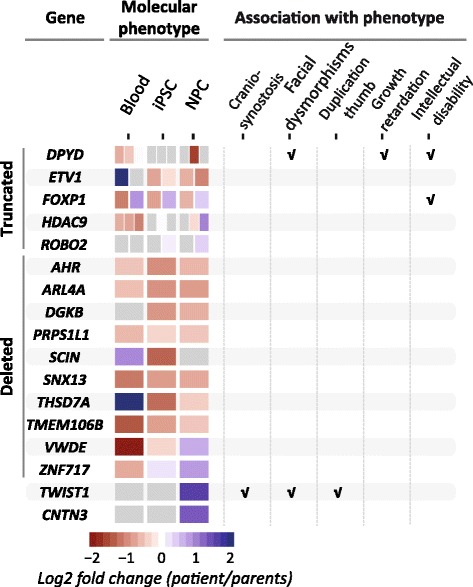
Overview of molecular phenotypes and their association with the patient’s phenotype. Selection of the genes located near the breakpoints with affected coding sequences and/or altered expression. The heatmap indicates the log2 fold expression differences between the patient and the parents in the three different cell types. Expression changes of the truncated genes are split into separate boxes for each gene fragment. *Grey boxes* are shown for genes with less than ten normalized read counts. More details are provided in Additional file 3: Table S2 and Additional file 4: Table S3

To identify molecular consequences of the chromothripsis rearrangements we performed RNA-seq on the iPSC lines and the iPSC-derived NPCs of the patient and the parents. We systematically analyzed the expression patterns of deleted genes, genes with disrupted coding sequences, and differentially expressed genes in close proximity to the breakpoints. Sixty-seven protein-coding genes are located across or within 1 Mb from the rearrangements (Fig. 2; Additional file 3: Table S2). Sixty (89%) of these are expressed in at least one of the samples. Ten genes are located on three deleted fragments (Fig. 3; Additional file 1: Figure S5). Four of these hemizygously deleted genes (*SNX13* (OMIM:606589), *TMEM106B* (OMIM:613413), *AHR* (OMIM:600253) and *ARL4A* (OMIM:604786)) show a consistent reduced expression in all patient’s samples compared to the parents’ samples (Fig. 3; Additional file 1: Figure S5). Although in theory the loss of these genes on the affected paternal alleles may have contributed to the patient’s phenotype through haploinsufficiency, none of these genes have previously been associated with any of the patient’s symptoms in the literature and were therefore considered unlikely to have played a major role in disturbing the development of the patient (Fig. 3; Additional file 4: Table S3).

### Expression-dependent molecular effects on broken genes

**Fig. 4:**
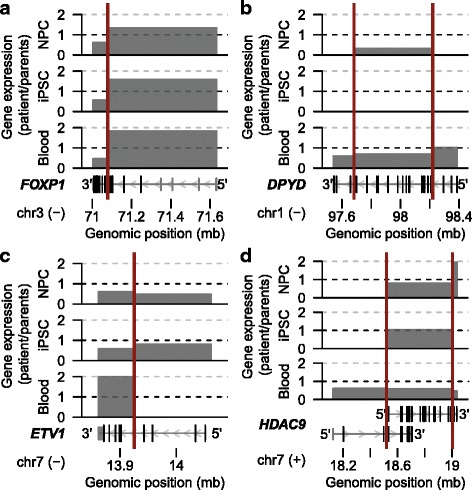
Altered expression patterns of genes with disrupted coding sequences. Relative expression differences of disrupted genes **a** *FOXP1* (NM_032682), **b** *DPYD* (NM_000110), **c** *ETV1* (NM_001163152), and **d** *HDAC9* (NM_001204144 and NM_178423) between the patient and parents in the iPSC-derived NPCs, iPSCs, and blood cells. Gene structures of the RefSeq transcripts described above are shown below the graphs. *Vertical red lines* indicate the breakpoint locations. *Minus* and *plus signs* indicate the DNA strand. Expression is not shown for fragments with less than ten normalized read counts in the patient or the parents

The coding sequences of six genes have been interrupted by the rearrangements (Fig. 4). Of these six disrupted genes, only *AGMO* (*TMEM195*) is not expressed in any of the assessed cell types. The 5′ part of *FOXP1* is fused to an inverted region on chromosome 7 containing parts of the *HDAC9* gene. The two disrupted genes are fused in opposite orientation and therefore do not directly form a fusion protein. However, we previously showed that there is read-through transcription from the 5′ part of *FOXP1* to the other strand of chromosome 7, leading to expression of a short fusion protein [19]. The 5′ fused part of *FOXP1* is expressed at higher levels in the cells derived from the patient in comparison with the cells of the parents (Fig. 4a). In contrast, the 3′ fragment of *FOXP1* shows a reduction in expression of 55% on average in the patient’s cells (Fig. 4a). The 3′ part of *ETV1* is fused to the 5′ part of *DPYD* and this *DPYD-ETV1* fusion gene shows strong expression in blood cells [19] but not in the iPSCs and iPSC-derived neural progenitors (Fig. 4b, c). The expression of *DPYD-ETV1* is driven by the activity of the *DPYD* promoter, which is strong in blood but low in iPSCs and neural progenitors. The unaffected maternal *ETV1* allele is only expressed in the iPSCs and iPSC-derived neural progenitors, but at the RNA level expression of this allele cannot completely compensate for the loss of the paternal allele in these cell types (Fig. 4c). Both *DPYD* and *HDAC9* are disrupted by two breakpoints, but these breakpoints only have a minor impact on the expression of these genes in the assessed cell types [19] (Fig. 4b, d).

Of these six disrupted genes, *FOXP1* (OMIM:605515) and *DPYD* (OMIM:612779) are associated with (neuro-)developmental disorders and may thus be relevant for the patient phenotype (Fig. 3; Additional file 4: Table S3). *FOXP1* is an essential transcription factor involved in the development of many tissues, including the brain [41]. Heterozygous disruptions of *FOXP1* have been found in several patients with neurodevelopmental disorders, including intellectual disability, autism spectrum disorder, and motor development delay [41]. *DPYD* encodes DPD (dihydropyrimidine dehydrogenase), an enzyme involved in the catabolism of pyrimidine bases [42]. Most carriers of heterozygous *DPYD* mutations are healthy, but some patients with hemizygous deletions affecting *DPYD* have neurodevelopmental disorders, including autism spectrum disorders [43–45], schizophrenia [46], epilepsy [47], and intellectual disability [42, 48, 49]. The disrupted coding sequences, altered expression, and association with congenital disease make it likely that the disruptions of *FOXP1* and possibly *DPYD* contributed to the developmental delay and intellectual disability of the patient. However, none of the broken or deleted genes have been associated with craniosynostosis, one of the major phenotypic appearances of the patient (Fig. 3; Additional file 4: Table S3).

### Overexpression of *TWIST1* and *CNTN3* in the patient’s iPSC-derived NPCs

**Fig. 5:**
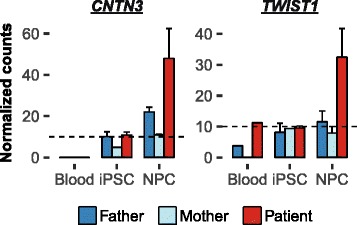
Overexpression of *TWIST1* and *CNTN3* exclusively detectable in the iPSC-derived neural progenitors. Bar graphs of *CNTN3* and *TWIST1* normalized gene expression in the blood cells, iPSCs, and iPSC-derived neural progenitors of the chromothripsis patient and the parents. The *dashed horizontal line* indicates the expression threshold of ten normalized read counts. *Error bars* indicate the standard error

Two genes that are located on inverted regions, but are not deleted or truncated, *TWIST1* and *CNTN3*, show a more than twofold difference in RNA expression in the NPCs derived from the patient in comparison to the parental cells (Fig. 5). Both genes are hardly expressed in blood cells and the coding sequences of these genes are not disrupted by the rearrangements, indicating that positional effects rather than gene dosage cause their misexpression. *CNTN3* (also known as *contactin-3*, *PANG*, or *BIG-1*) is a member of the contactin family of neural cell adhesion molecules, but little is known about the specific functions of *CNTN3* [50–52]. *CNTN3* is mainly expressed postnatally in specific subsets of neurons and promotes neurite outgrowth in isolated rat neurons [52, 53]. Copy number changes of close family members *CNTN4*, *CNTN5*, and *CNTN6* have been associated with autism spectrum disorders [54, 55]. We hypothesized that misexpression of *CNTN3* in neural progenitor cells may have affected the proper differentiation and migration of the patient’s cortical neurons. To test this hypothesis we performed in utero electroporations of *CNTN3* overexpression plasmids in neural progenitors of the developing mouse cortices. In this experiment we did not detect any change in the migration of neurons in the cortical layers (Additional file 1: Figure S6). We therefore consider it unlikely that misexpression of *CNTN3* has interfered with this developmental process in the patient.

### Deregulation of *TWIST1* associated with patient’s phenotype

The other overexpressed gene located near the breakpoints in the patient NPCs is *TWIST1*, a basic helix-loop-helix (bHLH) factor essential for mesoderm and neural crest development, including the morphology and migration of head mesenchyme cells [56]. *TWIST1* mutations and deletions (OMIM: 601622) are the main cause of Saethre–Chotzen syndrome (OMIM: 101400), characterized by craniosynostosis and limb abnormalities, including polydactyly, brachydactyly, and syndactyly [57, 58]. Several craniosynostosis patients with translocation breakpoints near *TWIST1* have been described [59–61]. The phenotypes of these patients largely resemble the phenotype of the patient described in this study. Overexpression of *TWIST1* has been shown to inhibit osteoblast differentiation in vitro and overexpression of *Twist1* in mouse embryonic limbs lead to reduced limb size [62–64]. Ectopic *TWIST1* expression may disturb the balance between *TWIST1*, its dimerization partners such as *HAND2* and *TCF12*, and its competitors for binding partners [65–67]. In general, however, the phenotypes of patients with *TWIST1* mutations and deletions are linked to *TWIST1* haploinsufficiency [58]. In addition, trisomy of the 7p15.3pter locus including the *TWIST1* gene has been associated with delayed closure of the fontanels, the opposite phenotype of the patient described in this study and patients with *TWIST1* haploinsuffiency [68, 69].

**Fig. 6:**
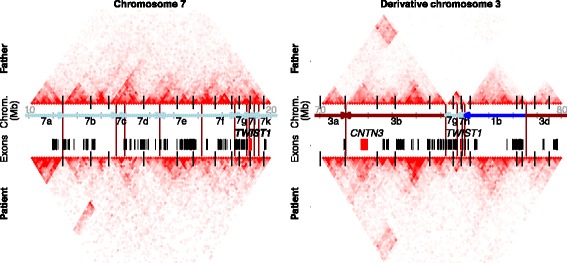
Gains of genomic interactions on the derivative chromosomes of the patient. Hi-C chromatin interaction maps of the patient’s (cell line UMCU15, *bottom panels*) and father’s (cell line UMCU23, *top panels*) chromosome 7 (*left panels*) and derivative chromosome 3 (*right panels*). Interactions are shown at 100-kb resolution. The *vertical black lines* at the bases of the heatmaps depict the predicted TAD boundaries in hESCs as determined by Dixon et al. [38]. *Vertical red lines* between the interaction maps indicate the breakpoint locations in the patient

The overexpression of *TWIST1* in the NPCs derived from the patient indicates a disturbed transcription regulation. We hypothesized that this deregulation may have led to decreased *TWIST1* expression in neural crest and mesodermal cell types, resulting in a phenotype parallel to that of patients who have haploinsufficiency of this gene. To test this hypothesis, we investigated the regulatory landscape surrounding the *TWIST1* gene. First we performed Hi-C to determine the genomic interactions on the derivative chromosomes in the patient. The topologically associated domain (TAD) structures of the unaffected chromosomes of the patient and father are similar to the previously published TAD structures by Dixon and colleagues [38] (Fig. 6; Additional file 1: Figure S7). Disruption of TAD boundaries can cause ectopic interactions between gene promoters and enhancers and this may lead to disease [16]. Thirteen TADs are directly affected by the breakpoints in the patient and five TAD boundaries are deleted (Fig. 6; Additional file 1: Figure S7). Many ectopic genomic interactions cross the breakpoint junctions on the derivative chromosomes of the patient. For example, many interactions between the genomic regions of chromosome 1, 3, and 7 that form derivative chromosome 3 in the patient are not present in the father (Fig. 6). We could not precisely discern between reads of the unaffected maternal and affected paternal alleles and therefore could not specifically determine the genomic architecture of the derivative chromosomes.

**Fig. 7:**
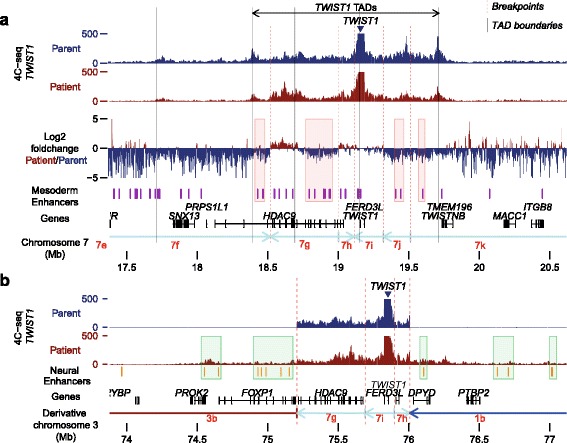
Gains and losses of enhancer interactions with the *TWIST1* locus in the patient. **a** 4C-seq data show that *TWIST1* mainly contacts a region encompassing three TADs (termed *TWIST1 TADs*) in the NPCs of the father (cell line UMCU23). The *y-axis* indicates the number of normalized 4C-seq reads cutoff at 500 normalized reads. TAD boundaries in H1-ESCs were determined by Hi-C analysis by Dixon et al. [38]. ChromHMM analysis of Roadmap ChIP-seq data of primary fibroblasts with high *TWIST1* expression indicates that these *TWIST1* TADs contain multiple enhancers active in mesodermal cells (shown in *purple*). The *TWIST1* 4C-seq data of the patient’s NPCs (UMCU15) shows that *TWIST1* has reduced interactions with several of these enhancers (*red highlights*), which likely had an impact on *TWIST1* expression in the patient. **b** The 4C-seq data, depicted on the derivative chromosome 3 in the patient, shows that *TWIST1* gained several ectopic contacts with enhancers active in neural cells in the patient. Enhancer activity was obtained from ChromHMM analysis of Roadmap ChIP-seq data of NPCs derived from differentiation of hESCs. 4C-seq using two of these enhancers as baits confirms the ectopic interactions between the enhancers and *TWIST1* (Additional file 1: Figure S8). These ectopic interactions may explain the overexpression of *TWIST1* in the patient’s NPCs

Secondly, we performed 4C-seq on the NPCs of the patient and the father using *TWIST1* as bait to determine potential gains and losses of genomic interactions of *TWIST1* in the patient. *TWIST1* mostly interacts with a region encompassing three putative TADs in the NPCs of the father (Fig. 7a). These three TADs are disrupted by five breakpoints in the patient and parts of these TADs are inverted or translocated away from *TWIST1*. These disrupted *TWIST1* TADs contain several mesodermal enhancers active in cells with high *TWIST1* expression and known *TWIST1* enhancers (Fig. 7a) [70–72]. The *TWIST1* 4C-seq shows that there are losses of interactions between these enhancers and *TWIST1* in the patient (Fig. 7a, red highlights). These losses of contacts with several of its enhancers could lead to reduced *TWIST1* expression in neural crest-derived cells involved in craniosynostosis and possibly contribute to the craniosynostosis phenotype [58].

In addition, the 4C-seq experiments show that *TWIST1* gained aberrant interactions with several enhancers active in neural progenitor cells (Fig. 7b, green highlights; Additional file 1: Figure S8). It is likely that these ectopic enhancer interactions drive the overexpression of *TWIST1* in the NPCs of the patient. Thus, chromosome conformation capture data suggest that *TWIST1* has lost interactions with mesodermal enhancers and has gained new interactions with enhancers that are active in neurons, which may explain deregulation of *TWIST1* expression in the patient. The resemblance with phenotypes of patients with *TWIST1* mutations, deletions, and translocations strongly suggests a causative role of the *TWIST1* deregulation in the development of the phenotype of our patient. This important molecular phenotype with a likely impact on the phenotype of the patient is only detectable in the patient iPSC-derived NPCs.

## Discussion

We determined the molecular effects of complex chromosomal rearrangements by transcriptome analyses on blood cells, iPSCs, and iPSC-derived neural progenitors from an MCA/MR patient with chromothripsis. In addition, we performed chromosome conformation capture analyses on the iPSC-derived neural progenitors to study the genomic architecture of the derivative chromosomes. We confirmed several previously identified direct effects of the breakpoints on gene expression, such as reduced expression of several hemizygously deleted genes and misexpression of fused (*DPYD-ETV1*) and truncated genes (*FOXP1* and *ETV1*) [19]. In addition, some genes that are located near the breakpoints but are not directly affected by the breakpoints (*TWIST1* and *CNTN3*) were differentially regulated in the patient, indicating effects of the rearrangements on the regulatory DNA landscape. The altered expression of *TWIST1*, loss of genomic interactions with several of its enhancers, and the resemblance of the patient phenotype with *TWIST1* ^*+/−*^ patients indicate that the *TWIST1* deregulation is a major cause of the patient phenotype. The effect on *TWIST1* expression was not detectable in the blood cells of the patient, highlighting the importance of using disease-relevant cell types for the interpretation of the consequences of genomic rearrangements.

Although genomic rearrangements caused by chromothripsis are non-recurrent, the effects of complex rearrangements on the phenotype of a patient may be inferred from patients with similar phenotypes caused by less complex genomic rearrangements. In this study, especially the detected deregulation of *TWIST1* expression, which was only detected in the patient iPSC-derived NPCs, may explain a large part of the patient phenotype (the craniosynostosis and the doubling of the thumbs). The coding sequence of *TWIST1* is not affected by the rearrangements, but translocations near *TWIST1* have been found before in patients with similar phenotypes [59–61]. Effects on *TWIST1* expression would have been difficult to predict by only studying the genomic variation of the patient, which demonstrates the importance of transcriptome analysis by RNA-seq to detect such effects in disease-relevant cell types. 4C-seq analyses showed that *TWIST1* gained and lost interactions with several enhancers, which could have led to the deregulation of the normal gene expression in different cell types. This example of *TWIST1* misexpression due to positional effects highlights the importance of not focusing solely on copy number changes or truncated and fused genes when studying the effects of chromosomal rearrangements [14]. This is further underscored by our finding that only half of the deleted genes in this patient show a consistent reduced expression, suggesting dosage compensation at the RNA level for the other half of the deleted genes. With our approach, we narrowed down a list of 67 candidate genes within 1 Mb of the breakpoints to a list of three genes that likely contribute to the patient’s phenotype.

Only a minority of the *TWIST1* ^*+/−*^ patients show signs of developmental delay and intellectual disability like those observed for the patient described in this study. It is very well possible that a combination of molecular effects led to the complex phenotype of the patient. For example, the disrupted *FOXP1* and *DPYD* genes are known MCA/MR genes that may have contributed to the intellectual disability and developmental delay in our patient. We cannot exclude that there are additional molecular effects in other cell types that also have contributed to the phenotype.

## Conclusions

By analyzing the transcriptomes of blood cells, iPSCs, and iPSC-derived neuronal cells of a chromothripsis patient and both parents we identified the functional effects of the rearrangements that likely have contributed to the patient’s phenotype. In particular we observed a cell type-specific effect of the rearrangements on the expression of *TWIST1*, even though the coding sequence of this gene was not disrupted by the rearrangements. This study shows the power of transcriptome and chromosome conformation capture analyses to detect effects of structural rearrangements on both coding sequences and regulatory elements. We identified clinically relevant molecular effects specific to the iPSC-derived neuronal cells. These findings underscore the importance of using disease-relevant cell types to better understand the molecular effects of chromosomal rearrangements.

## Additional files

Additional file 1: Document containing all supplemental figures and legends. (PDF 30908 kb)
Additional file 2: Table S1. Primer sequences used for each viewpoint in the 4C-seq experiments. Primer sequences are shown without the sequences of the sequencing primer and the P5 and P7 Illumina adaptors. (XLSX 10 kb)
Additional file 3: Table S2. Normalized RNA expression values for all 67 protein-coding genes located near the breakpoints. Expression levels of fragments of genes disrupted by the chromothripsis breakpoints are shown separately. Log2 fold expression changes between the patient and parents of more than 1 or less than −1 and *p* values of less than 0.05 are highlighted in *red*. Differential expression was calculated using DESeq nbinomTest. (XLSX 31 kb)
Additional file 4: Table S3. Associations of genes located near the breakpoints with human and mouse disease phenotypes. Associations of the genes with human disease phenotypes were obtained from the MalaCards human disease database (http://www.malacards.org/; only phenotypes with a score of more than 1 are included). Mouse embryonic expression data were retrieved from the Gene Expression Database (GXD) from Mouse Genome Informatics (http://www.informatics.jax.org/expression.shtml) and the eMouseAtlas (http://www.emouseatlas.org/). Phenotypes of homozygous mouse knockouts were obtained from Mouse Genome Informatics (http://www.informatics.jax.org/). Data were not available for all genes. (XLSX 23 kb)

## Abbreviations

FBS: Fetal bovine serum
hESC: Human embryonic stem cell
IL: Interleukin
iPSC: Induced pluripotent stem cell
Mb: megabase
MCA/MR: Multiple congenital abnormalities and/or mental retardation
NPC: Neural progenitor cell
PBMC: Peripheral blood mononuclear cell
PBS: Phosphate-buffered saline
RT: Room temperature
TAD: Topologically associated domain
TPO: Thrombopoietin.
