# Supplementary material for: Molecular dissection of germline chromothripsis in a developmental context using patient-derived iPS cells
Source: Genome Med. 2017 Jan 26;9:9. doi: 10.1186/s13073-017-0399-z (PMC5270341; doi:10.1186/s13073-017-0399-z)
Supplement: Additional file 1: — Document containing all supplemental figures and legends. (PDF 30908 kb) [file 13073_2017_399_MOESM1_ESM.pdf]

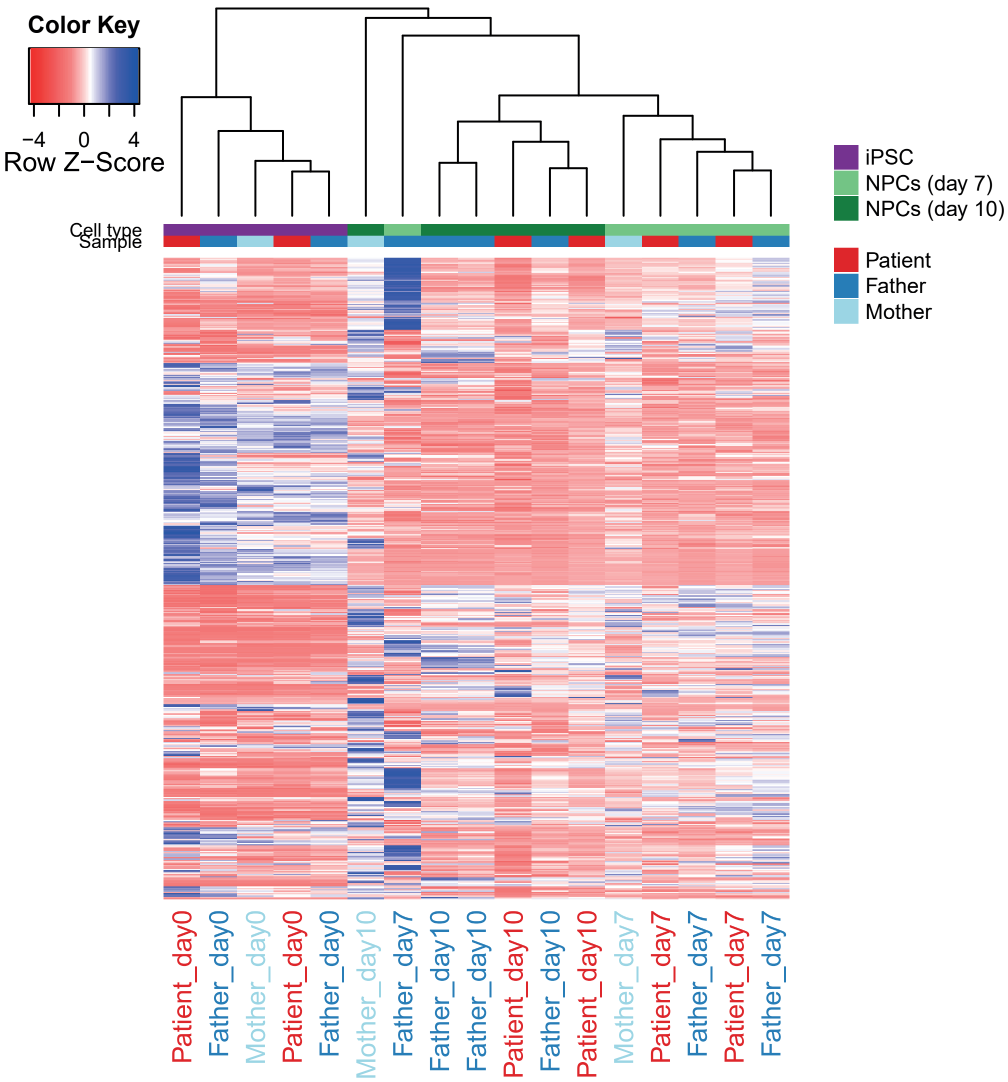

Supplemental Fig. S1: RNA expression profiles of day 7 and day 10 NPCs cluster together. Heatmap showing the results of an Euclidean hierarchical clustering analysis of the 500 genes with highest variance between the 17 samples.

a

Cell line		Number of metaphases investigated	Banding quality		Karyotype
			Best	Average	
**UMCU14**	Patient	20	500	300	46,XX,der(1)(7pter→7p21.3::7p21.3→7p21.2::1p21.3→1qter),<br>der(3)(3pter→3p13::3p12.3→3p13::7p21.1→7p21.1::1p21.3→1p21.3::3p12.3→3qter),<br>der(7)(12qter→12q14.2::7p21.1→7p21.1::7p21.1→7qter),<br>der(12)(12pter→12q14.2::7p21.2→7p21.1::1p21.3→1pter)
**UMCU15**	Patient	20	500	400	47,XX,der(1)(7pter→7p21.3::7p21.3→7p21.2::1p21.3→1qter)x2,<br>der(3)(3pter→3p13::3p12.3→3p13::7p21.1→7p21.1::1p21.3→1p21.3::3p12.3→3qter),<br>der(7)(12qter→12q14.2::7p21.1→7p21.1::7p21.1→7qter),<br>der(12)(12pter→12q14.2::7p21.2→7p21.1::1p21.3→1pter)
**UMCU16**	Patient	20	500	400	46,XX,der(1)(7pter→7p21.3::7p21.3→7p21.2::1p21.3→1qter),<br>der(3)(3pter→3p13::3p12.3→3p13::7p21.1→7p21.1::1p21.3→1p21.3::3p12.3→3qter),<br>der(7)(12qter→12q14.2::7p21.1→7p21.1::7p21.1→7qter),<br>der(12)(12pter→12q14.2::7p21.2→7p21.1::1p21.3→1pter)
**UMCU23**	Father	20	400	400	46,XY
**UMCU32**	Father	20	400	400	46,XY
**UMCU30**	Mother	21	400	400	46,XX,der(20)t(1;20)(q25;p11.2 or q11.2)

b

UMCU15

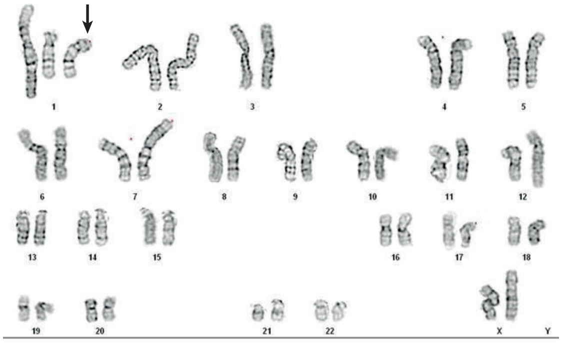

c

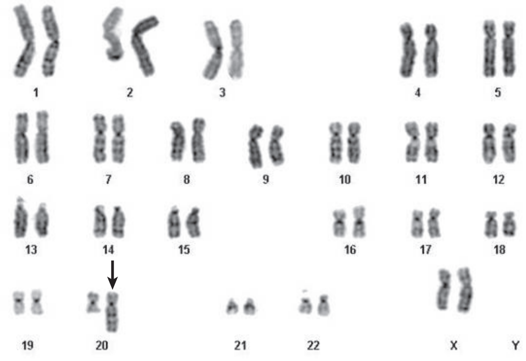

UMCU30

Supplemental Fig. S2 Karyotypes of the patient's and parent's iPSC lines. **a** Overview of karyotyping results. Most iPSC lines contained the expected karyotypes. One iPSC line derived from the patient and one derived from the mother acquired an additional genomic rearrangement during cultivation. **b** Karyogram of one of the patient iPSC lines (UMCU15) containing a duplication of derivative chromosome 1. **c** Karyogram of the iPSC line derived from the mother (UMCU30) showing a translocation between chromosome 20 and a fragment of chromosome 1. This rearrangement is located more than 70 Mb away from the locations of the rearrangements in the patient.

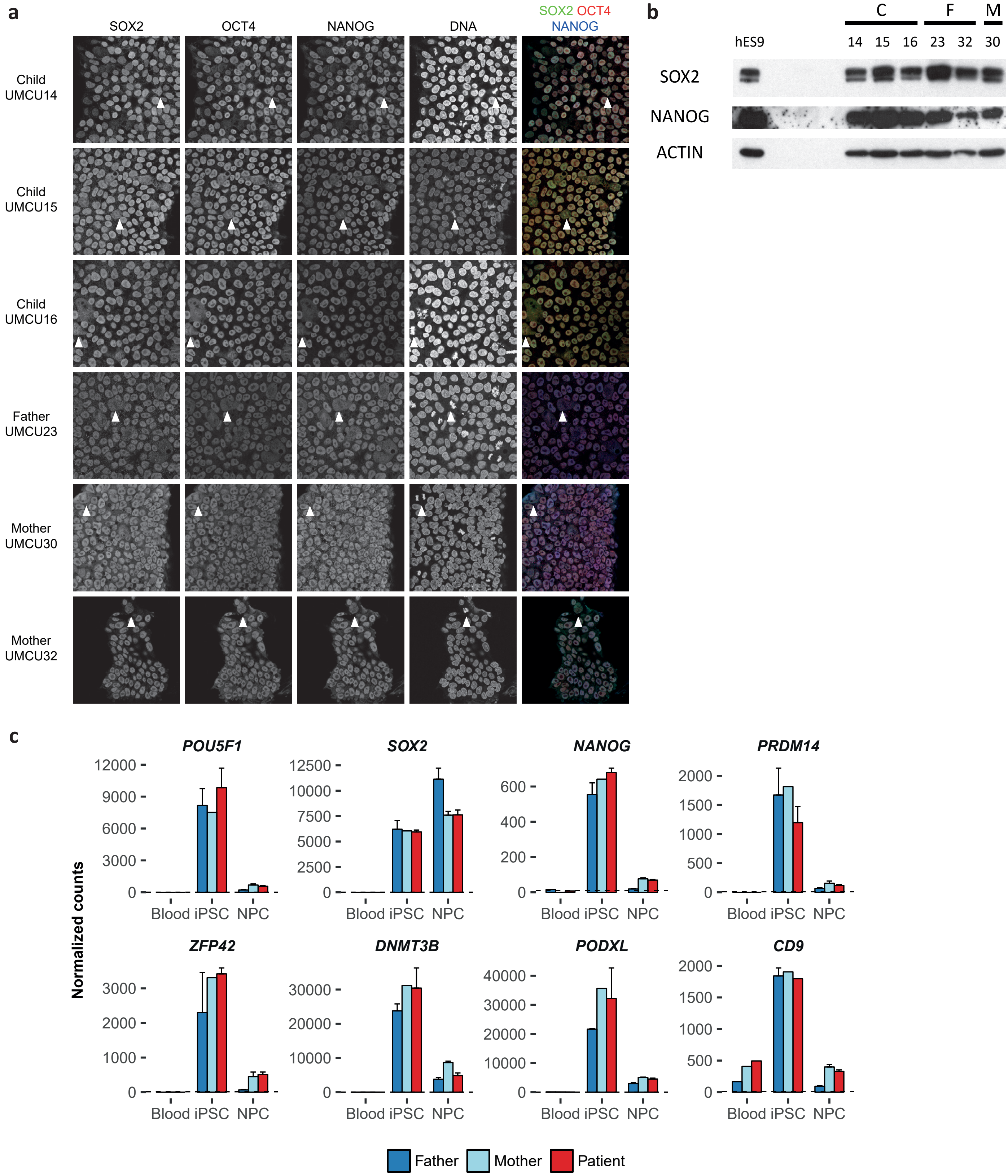

Supplemental Fig. S3 Patient's and parental iPSC lines express high levels of pluripotency markers. **a** Immunostainings showing the expression of the pluripotency markers SOX2, OCT4 and NANOG (column 1 to 3). DNA is stained with DAPI (fourth column). **b** Western blot showing expression of SOX2, NANOG and ACTIN in hES9 cells (positive control) and the patient/child (14, 15, 16), father (23 and 32) and mother (30). The iPSC lines show expression levels of SOX2 and NANOG comparable to the hES9 cell line. **c** Bar graphs showing the normalized RNA expression of eight genes associated with pluripotency. Patient's RNA expression data was generated for cell line UMCU14 and UMCU15. SOX2 is a marker for both pluripotent stem cells and early neural cells. The dashed horizontal line indicates the expression threshold of 10 normalized read counts. Error bars indicate standard error.

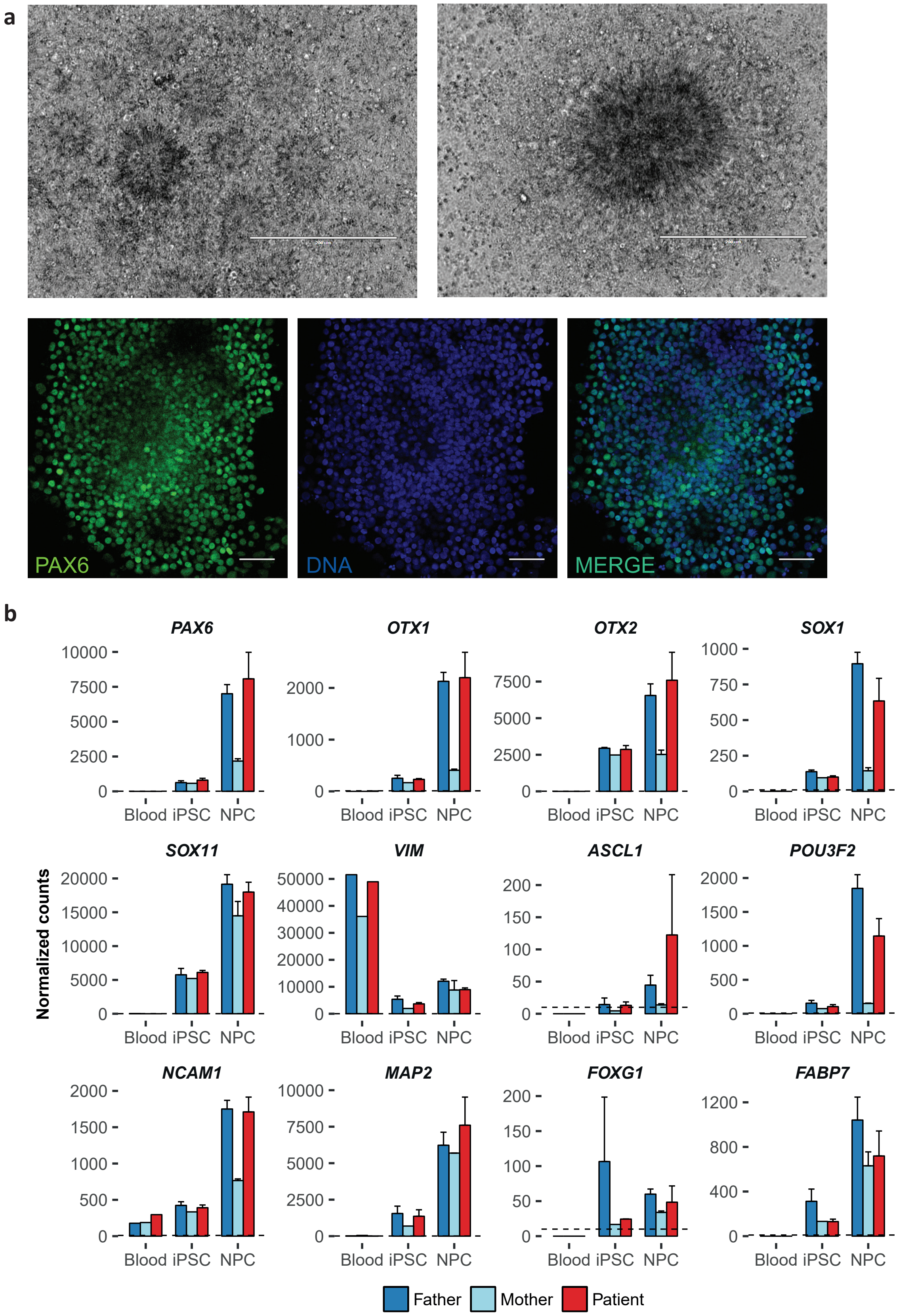

Supplemental Fig. S4 iPSC-derived NPCs form neural rosettes and express high levels of early neural markers. **a** Examples of brightfield (top) and immunofluorescent images (bottom) of neural rosettes formed by iPSC-derived NPCs 15 days after the start of differentiation. PAX6 is a marker for NPCs (bottom, left). DNA is stained with DAPI (bottom, center). The scale bars in the upper images indicate 200 µm. **b** Bar graphs showing the normalized expression of eight genes associated with early neural cells in patient's and parental blood cells, iPSCs and iPSC-derived NPCs. The dashed horizontal line indicates the expression threshold of 10 normalized read counts. Error bars indicate standard error.

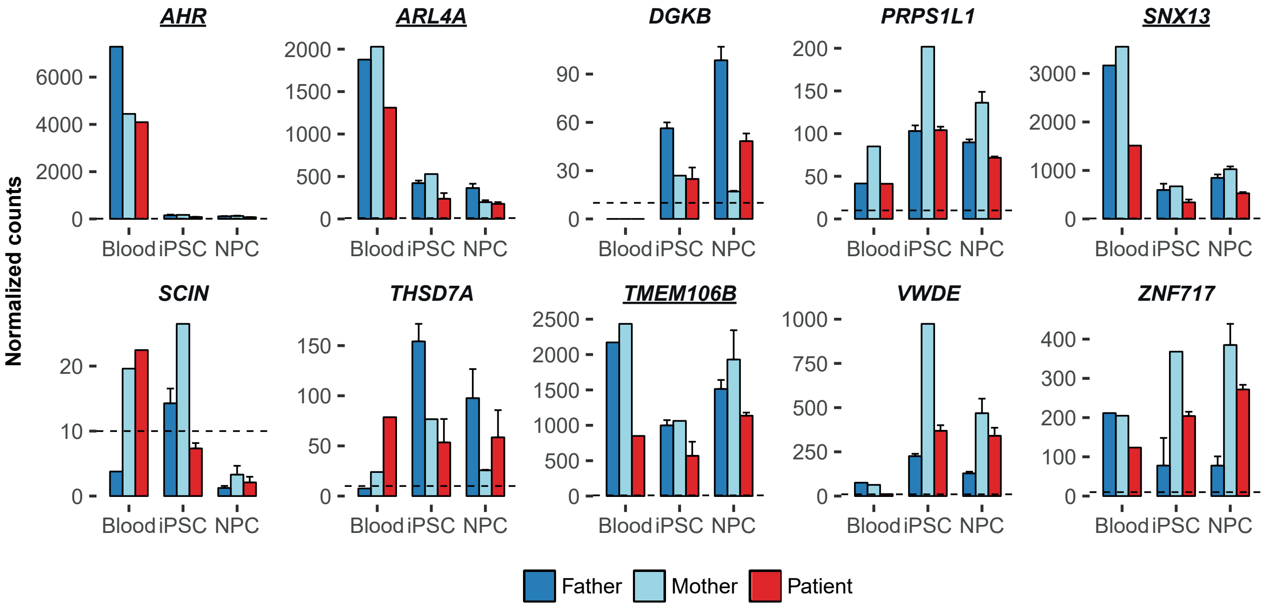

Supplemental Fig. S5 Decreased RNA expression of four of the ten deleted genes in the patient. Bar graphs showing the normalized RNA expression for the ten deleted genes located on four deleted fragments in the patient. The four genes with underscored names show a decreased expression in all three cell types. The dashed horizontal line indicates the expression threshold of 10 normalized read counts. Error bars indicate standard error.

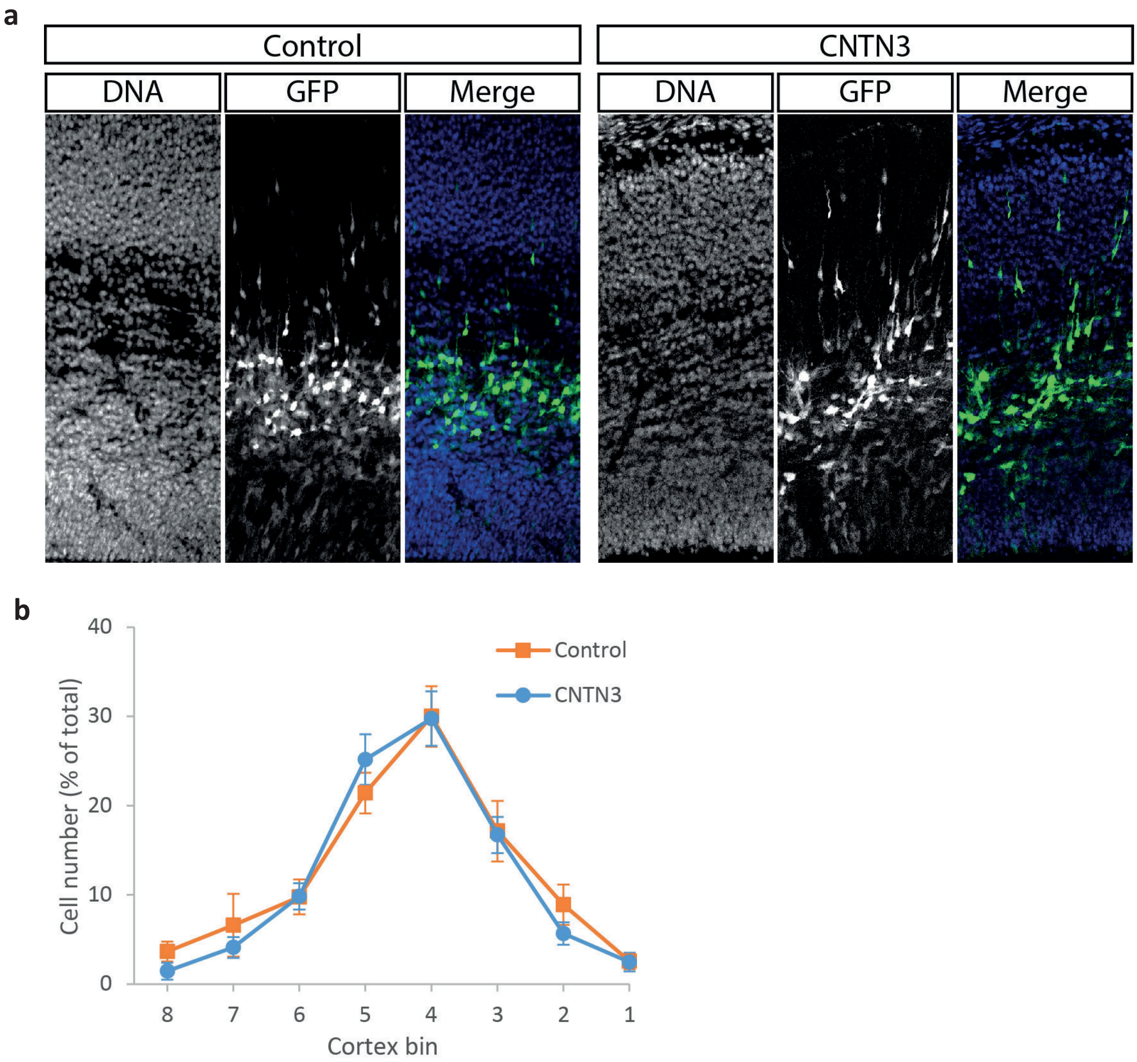

Supplemental Fig. S6 Migration of E16.5 mouse embryonic cortical neurons not affected by CNTN3 overexpression. **a** Immunofluorescent stainings of brain sections of a E16.5 mouse embryo treated with control (left) or pCAG-*CNTN3* vectors (right) on day E14.5. A pCAG-GFP vector was co-injected with the pCAG-*CNTN3* or control constructs to identify successfully targeted cells. DNA was stained with Hoechst. **b** Quantification of the number of GFP-positive cells in equally sized bins covering the cortical layers from the ventricle border (bin 1) to the pial surface (bin 8).

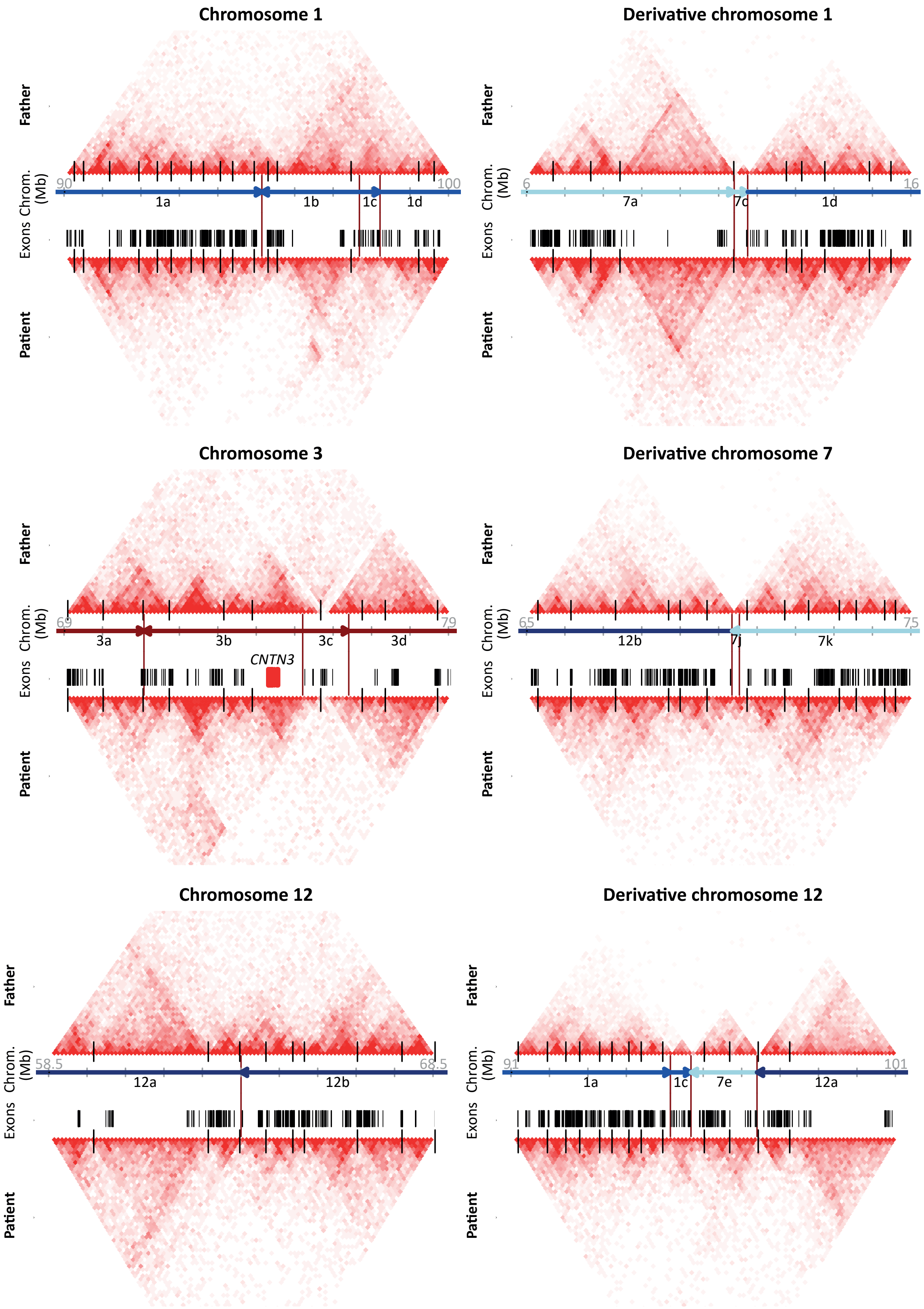

Supplemental Fig. S7: Changes in genomic interactions on derivative chromosomes of the patient. Hi-C chromatin interactions maps of the patient's (UMCU15) and father's (UMCU23) chromosomes (left panels) and derivative chromosomes (right panels). Interactions are shown at a 100 kb resolution. The vertical black lines at the bases of the heatmaps depict the predicted TAD boundaries in hESCs as determined by Dixon et al [59]. Vertical red lines between the interaction maps indicate the breakpoints locations in the patient.

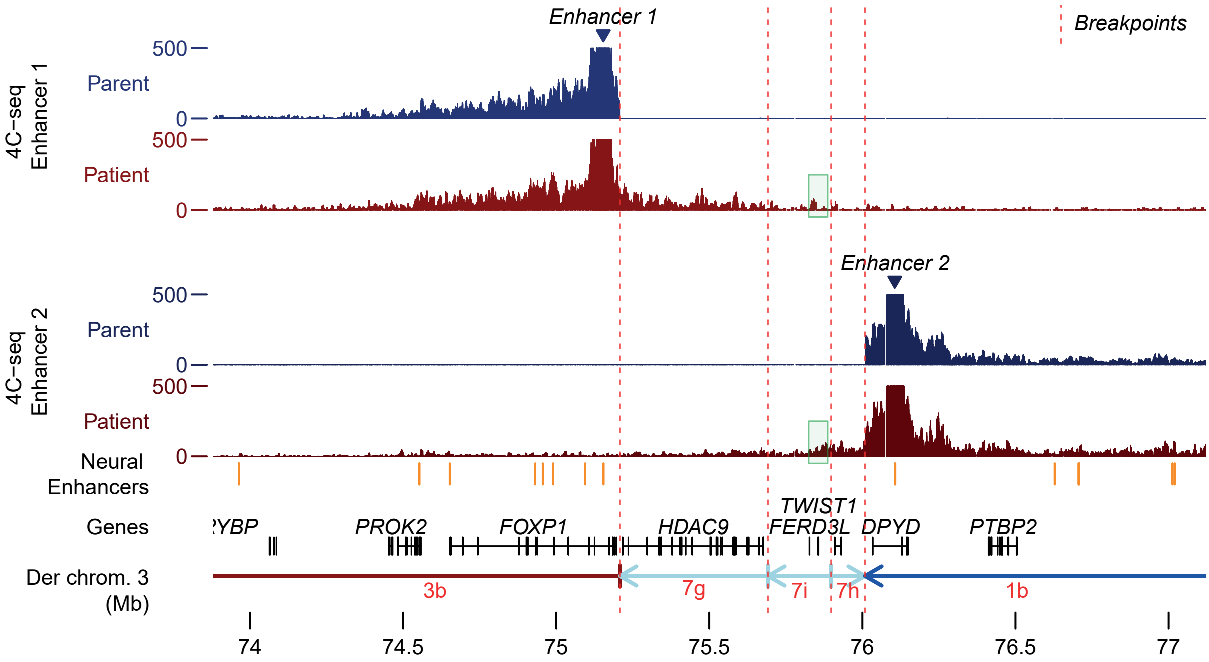

Supplemental Fig. S8 Enhancers active in NPCs gained ectopic interactions with *TWIST1* (highlighted in green) in the patient (UMCU15). Two enhancers active in NPCs (determined by ChromHMM analysis of Roadmap ChIP-seq data of hESC-derived NPCs) were used as bait for 4C-seq. These aberrant interactions with neural enhancers may be the cause of *TWIST1* overexpression in the patient's NPCs. The y-axes indicate the number of normalized 4C-seq reads cut-off at 500 reads.
